# Causality and Information Transfer Between the Solar Wind and the Magnetosphere–Ionosphere System

**DOI:** 10.3390/e23040390

**Published:** 2021-03-25

**Authors:** Pouya Manshour, Georgios Balasis, Giuseppe Consolini, Constantinos Papadimitriou, Milan Paluš

**Affiliations:** 1 Department of Complex Systems, Institute of Computer Science of the Czech Academy of Sciences, Pod Vodárenskou věží 2, 182 07 Prague 8, Czech Republic; manshour@cs.cas.cz; 2 Institute for Astronomy, Astrophysics, Space Applications and Remote Sensing, National Observatory of Athens, I. Metaxa & Vas. Pavlou Street, 15236 Penteli, Greece; gbalasis@noa.gr (G.B.); constantinos@noa.gr (C.P.); 3 INAF-Istituto di Astrofisica e Planetologia Spaziali, 00133 Rome, Italy; giuseppe.consolini@iaps.inaf.it; 4 Department of Physics, National and Kapodistrian University of Athens, Panepistimiopolis, Zografos, 15784 Athens, Greece; 5 Space Applications & Research Consultancy, SPARC P.C., 10551 Athens, Greece

**Keywords:** time series, causality, information transfer, time reversal, solar wind-magnetosphere–ionosphere system, space weather

## Abstract

An information-theoretic approach for detecting causality and information transfer is used to identify interactions of solar activity and interplanetary medium conditions with the Earth’s magnetosphere–ionosphere systems. A causal information transfer from the solar wind parameters to geomagnetic indices is detected. The vertical component of the interplanetary magnetic field (Bz) influences the auroral electrojet (AE) index with an information transfer delay of 10 min and the geomagnetic disturbances at mid-latitudes measured by the symmetric field in the H component (SYM-H) index with a delay of about 30 min. Using a properly conditioned causality measure, no causal link between AE and SYM-H, or between magnetospheric substorms and magnetic storms can be detected. The observed causal relations can be described as linear time-delayed information transfer.

## 1. Introduction

One of the fundamental problems in space weather studies is the way the Earth’s magnetosphere–ionosphere system responds to the solar activity and to interplanetary medium conditions [1]. In fact, due to the continuous transfer of energy from solar wind as an external driver into the magnetosphere–ionosphere system along with different internal processes operating in various spatial and temporal scales [2,3,4,5,6], one faces the emergence of complex dynamics. This asserts that such a system should be considered as a non-equilibrium [7] complex system consisting of different coupled subsystems. Investigating and understanding such couplings is of paramount importance for providing a reliable prediction of the space weather [8], and thus the geomagnetic observables play an important role, among which magnetic storms and magnetospheric substorms are of particular interest.

Geomagnetic storms and substorms are two manifestations of the solar wind– magnetosphere–ionosphere (SMI) interactions, which are related to different sets of phenomena occurring in different regions of the near-Earth plasma environment [9,10,11]. The main difference between these two response modes of the SMI dynamics stands in the different magnetospheric regions and currents involved in them. Indeed, the term geomagnetic substorm generally refers to an enhancement of the energy/particle deposition rate in the high-latitude Earth’s ionosphere, due to the increase of the auroral electrojet currents and of the field-aligned currents (FACs), which transfer plasma from the mid equatorial regions of the Earth’s magnetospheric tail to the polar ionosphere. The geomagnetic/auroral substorms are mainly impulsive phenomena, which are the consequence of two different magnetospheric phenomena, the enhancement of the magnetospheric large scale convection due to the southward turning of the North–South component of the interplanetary magnetic field (IMF) and the occurrence of loading-unloading process in the Earth’s magnetospheric tail. These phenomena can be triggered by different IMF and solar wind changes and are generally characterized by strong activity bursts characterized by a typical time scale < 100 min. Differently, geomagnetic storms are related to a set of processes occurring at low latitudes and involving the inner region of the Earth’s magnetosphere. Indeed, a geomagnetic storm is related to the increase of a quasi-annular current system, the Ring Current, flowing on the equatorial region of the Earth’s magnetosphere at a distance between 2.5–3 $R_{E}$ and 9 $R_{E}$, where $R_{E}$ is the Earth radius. The enhancement of ring current occurs on longer times scales in comparison with the current systems involved in geomagnetic substorms and requires that the global magnetospheric convection persists for a longer time. Thus, the geomagnetic storms are generally associated with stable southward IMF conditions, which last for several hours. Another feature of geomagnetic storms is the duration, which can extend to several days.

The monitoring of these two phenomena can be done by measuring the geomagnetic disturbances generated by the enhancement of the current systems associated to them, i.e., the auroral electrojet current system and the ring current. The geomagnetic indices AE and SYM-H are two indices constructed to monitor the auroral electrojet current and the ring current by measuring the variation of the horizontal component (H) of the Earth’s magnetic field as observed on ground [12,13]. In other words, these two indices provide proxies of the enhancement of the current.

Understanding the interactions between magnetic storms and magnetospheric substorms has been one of the most challenging problems in space physics [14]. Indeed, the presence of any direct relationship between substorms and storms has been a great debate in recent years. Historically, the accumulation of successive substorms was considered as the main reason for the occurrence of storms [15]. However, several studies have shown that this may not be the case [10,11,14]. In this respect, detecting a correct causal relationship between various quantities is needed.

Among a huge number of formalisms to find and investigate causality relations among different time series, the information-theoretic approach has proved itself as a powerful framework to detect causal information flows in complex systems. In this approach, by assuming the presence of an information transfer between two coupled subsystems that can interact with each other, one tries to find an appropriate measure in order to extract the pure direction of the information flow and causality. In recent years, a number of information-theoretic measures have been proposed to uncover the underlying dynamics of interactions between the magnetosphere system and solar wind as well as the purely internal processes in this system [16,17,18,19,20,21,22]. For example, it has been proposed in [17] that during small geomagnetic disturbances a dominant flow of information exists from the geomagnetic activity indices AL as a substorm index into the symmetric horizontal component disturbances SYM-H as a magnetic storm index, using the bivariate transfer entropy [23]. Runge et al. [21] questioned the presence of any direct or indirect dependency between substorms and storms and by using a multivariate information-theoretic causality measure based on graphical models [24,25], they suggested that the statistical association between storms and substorms can be due to the presence of the common solar drivers. Recently, Stumpo et al. [22] have investigated the information flow between the solar wind parameters as well as geomagnetic indices, using the transfer entropy. They have shown that there is a strong information transfer from the vertical component of the interplanetary magnetic field Bz into the geomagnetic indices, with time delays of about 30 to 60 min. Moreover, they represented that substorms drive the storms due to the observed strong information flow from the AE into SYM-H index, which is in contrast with the results of Runge et al. [21].

In this paper, the information-theoretic approach to causality detection–conditional mutual information, also known as transfer entropy, which generalizes the Granger causality concept for nonlinear systems, as well as two independent recently developed approaches to causality, are used in order to identify interactions of solar activity and interplanetary medium conditions with the Earth’s magnetosphere–ionosphere systems. A unidirectional causality or information flow from the solar wind parameters to geomagnetic activity indices is detected and information transfer delays are identified. Although uncovered using a nonlinear causality method, the observed causal relations are described as a linear time-delayed information transfer. This assertion is supported using linear versions of three independent causality detection methods.

## 2. Data Description

**Figure 1:**
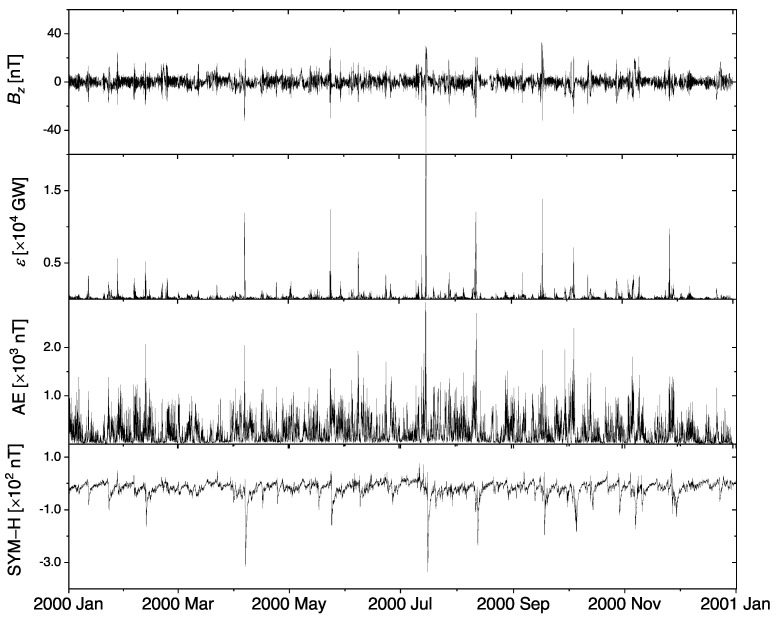
One year period (2000) of (from top to bottom) the vertical component of the interplanetary magnetic field Bz and solar wind–magnetosphere coupling parameter ϵ along with the geomagnetic activity indices of auroral electrojet (AE) and the symmetric field in the H component (SYM-H), with clear, strong activities. Time points with missing values are excluded from the analysis.

In this work, we focus on the year 2000 (from 1 January to 31 December), which corresponds to the maximum phase of solar cycle 23. In fact, this chosen time span consists of a number of geomagnetic storms and substorms, as observed in Figure 1.

The original 1-min time resolution data were downsampled to 5-min time resolution data for solar wind parameters as well as geomagnetic activity indices, similar to the work of Stumpo et al. [22]. We consider the vertical component of the interplanetary magnetic field Bz (downloaded from http://cdaweb.gsfc.nasa.gov/ (accessed on 16 April 2020)) and the energy coupling function ϵ between the solar wind and the magnetosphere [26], which are related to the energy–mass–momentum transfers from the interplanetary space to the near-Earth electromagnetic environment [1,16,27]. We calculate the Perreault–Akasofu coupling function ϵ using Equations (1) and (2) given in Stumpo et al. [22]. To investigate the geomagnetic activity, we use two well-known indices representing the auroral electrojet and the magnetospheric ring current, i.e., AE as a substorm index [12], and the SYM-H as a storm index [28] (both indices are downloaded from http://wdc.kugi.kyoto-u.ac.jp/ (accessed on 16 April 2020)).

## 3. Overview of Methods

### 3.1. Measuring Dependence with Mutual Information

As we mentioned above, the information-theoretic framework has proven itself as a powerful approach for the study of exchanging information among coupled time series. The information content of a discrete random variable *X* with a set of values Ξ is obtained by the *Shannon entropy* H(X) [29], defined as

(1) $$H\left(X\right)=-\sum_{x\in \Xi }p\left(x\right)\log p\left(x\right),$$

where p(x)= Pr{X=x}, x∈Ξ is the probability distribution function (PDF) of *X*. Note here that the entropy and information are usually measured in bits if the base of the logarithms in their definitions is 2, here we use the natural logarithm and therefore the units are called nats. By taking into account another discrete random variable *Y* with the set of values Υ, and PDF of p(y), the *joint entropy* H(X,Y) is defined in a similar way as

(2) $$H\left(X,Y\right)=-\sum_{x\in \Xi }\sum_{y\in \Upsilon }p\left(x,y\right)\log p\left(x,y\right).$$

where p(x,y) is the joint PDF of *X* and *Y*. The joint entropy can also be expressed in terms of *conditional entropy* H(Y|X) of *Y* given *X* as H(X,Y)=H(Y|X)+H(X), which is easily defined as

(3) $$H\left(Y|X\right)=-\sum_{x\in \Xi }\sum_{y\in \Upsilon }p\left(x,y\right)\log p\left(y|x\right).$$

where p(y|x) denotes the conditional probability of *Y* given *X*. The average amount of common information, contained in two variables *X* and *Y*, is obtained by the *mutual information* I(X;Y), defined as

(4) I(X;Y)=H(X)+H(Y)−H(X,Y).

Therefore, by substituting Equations (1) and (2) into (4), one can simply find

(5) $$I\left(X;Y\right)=\sum_{x\in \Xi }\sum_{y\in \Upsilon }p\left(x,y\right)\log\frac{p(x,y)}{p(x)p(y)},$$

which is the averaged value of $\log\frac{p(x,y)}{p(x)p(y)}$. In fact, if two variables *X* and *Y* are independent, i.e., p(x,y)=p(x)p(y) then the mutual information I(X;Y) vanishes. This means that I(X;Y) can be considered as a general measure of dependence between two variables *X* and *Y*. The presence of any dependence among *X* and *Y* results in I(X;Y)>0; however, I(X;Y) is symmetric under the exchange of variables *X* and *Y* and thus cannot be used as a proper causality measure.

Consider now *n* discrete random variables $X_{1},\ldots ,X_{n}$ with values $\left(x_{1},\ldots ,x_{n}\right)\in \Xi_{1}\times \ldots \times \Xi_{n}$, with PDF’s $p(x_{i})$ for individual variables $X_{i}$ and the joint distribution $p(x_{1},\ldots ,x_{n})$. The mutual information $I(X_{1};X_{2};\ldots ;X_{n})$, quantifying the common information in the *n* variables $X_{1},\ldots ,X_{n}$ can be defined as

(6) $$I\left(X_{1};X_{2};\ldots ;X_{n}\right)=H\left(X_{1}\right)+H\left(X_{2}\right)+\ldots +H\left(X_{n}\right)-H\left(X_{1},X_{2},\ldots ,X_{n}\right).$$

It is possible, however, to define mutual information functionals quantifying common information of groups of variables and also various multivariate generalizations of the conditional mutual information, see Reference [30].

All the information-theoretic functionals can be defined for continuous random variables. The sums are substituted by integrals and the PDF’s by the probability distribution densities [31,32]. Among the continuous probability distributions a special role is played by the Gaussian distribution. Let $X_{1},\ldots ,X_{n}$ be an *n*-dimensional normally distributed random variable with a zero mean and an n×n covariance matrix $C=\{c_{ij}\}$. Then (see References [30,32] and references therein)

(7) $$I_{G}\left(X_{1};\ldots ;X_{n}\right)=\frac{1}{2}\;\sum_{i=1}^{n}\log\left(c_{ii}\right)\;-\;\frac{1}{2}\;\sum_{i=1}^{n}\log\left(\sigma_{i}\right),$$

where $c_{ii}$ are the diagonal elements (variances) and $\sigma_{i}$ are the eigenvalues of the covariance matrix C.

### 3.2. Inference of Causality and Time-Delayed Information Transfer

A common information-theoretic functional used for the causality detection is the *conditional mutual information* (CMI) I(X;Y|Z) of the variables *X* and *Y* given the variable *Z*, defined as

(8) I(X;Y|Z)=H(X|Z)+H(Y|Z)−H(X,Y|Z).

Obviously, if *Z* is independent of *X* and *Y*, then I(X;Y|Z)=I(X;Y). The CMI of Equation (8) can be rewritten in terms of mutual information measures as

(9) I(X;Y|Z)=I(X;Y;Z)−I(X;Z)−I(Y;Z),

where I(X;Y;Z)=H(X)+H(Y)+H(Z)−H(X,Y,Z). This indicates that I(X;Y|Z) characterizes the “net” dependence between *X* and *Y* without a possible influence of another variable, *Z*.

All multivariate information-theoretic functionals described above investigate simultaneous shared/conditioned information content among variables. However, in many real-world situations, this could occur with a time delay τ. This means that one may find $I\left(X(t);Y(t)\right)=0$ only due to the presence of a time delay between two processes *X* and *Y*. To be able to discover such a coupling correctly, one can modify the mutual information, and define a time-delayed mutual information as $I\left(X(t);Y(t+\tau )\right)$, which measures the average amount of information contained in the process *X* about the process *Y* in its future τ time units ahead. However, this measure could also contain information about the τ-future of the process *Y* contained in this process itself, if the processes *X* and *Y* are not independent, i.e., if I(X;Y)>0. In order to obtain the “net” information about the τ-future of the process *Y* contained in the process *X* we use the conditional mutual information $I\left(X(t);Y(t+\tau )|Y(t)\right)$, which was used by Paluš et al. [33] to define the coarse-grained transinformation rate, able to detect direction of coupling of unidirectionally coupled dynamical systems. In fact, this measure was proposed as a nonlinear generalization of the Granger causality. Based on the idea of finite-order Markov processes, Schreiber [23] introduced a “transfer entropy”, which is an equivalent expression for the time-delayed conditional mutual information [34,35]. Finally, the transfer entropy and CMI in the form, defined below, are equivalent to the Granger causality for Gaussian processes [36].

In a physical system, one usually deals with time series {x(t)} and {y(t)} as realizations of stochastic processes {X(t)} and {Y(t)}, respectively. In other words, if the processes {X(t)} and {Y(t)} are substituted by dynamical systems evolving in measurable spaces of dimensions *m* and *n*, respectively, the variables *x* and *y* should be considered as the components of m− and n−dimensional vectors. In empirical experiments, however, usually only one possible dimension of the phase space is known for each system. In this situation, a widely useful approach to estimate other unknown variables is phase space reconstruction, using the time delay embedding vectors according to Takens [37]. In this respect, an *m*-dimensional state vector *X* can be reconstructed as X(t)={x(t),x(t−η),…,x(t−(m−1)η)}, where η is the backward time-lag that can be set according to the embedding construction procedure based on the first minimum of the mutual information [38], in order to assure that different coordinates of the reconstructed state vector X(t) are sufficiently independent of each other.

Accordingly, time-delayed CMI defined above can be represented by

(10) $$I\left(X(t);Y(t+\tau )|Y(t)\right)=\;I\left(\left(x(t),x(t-\eta ),\ldots ,x(t-(m-1)\eta )\right);y\left(t+\tau \right)|\left(y(t),y(t-\rho ),\ldots ,y(t-(n-1)\rho )\right)\right),$$

where η and ρ are time-lags used for the embedding of the trajectories X(t) and Y(t), respectively. Formally, also Y(t+τ) should be expanded as y(t+τ),x(t+τ−ρ),…,y(t+τ−(n−1)ρ; however, only information about one component y(t+τ) in the τ-future of the system *Y* is used for simplicity. On the other hand, extensive numerical experience [35] suggests that the conditional mutual information in the form

(11) $$I\left(x(t);y(t+\tau )|y(t),y(t-\rho ),\ldots ,y(t-(n-1)\rho )\right)$$

is sufficient to infer coupling direction between the systems X(t) and Y(t). Here x(t) represents the present state of the cause variable (system) *X* in the present time *t* and y(t+τ) the future (“predicted”) value of the effect variable (system) *Y* in the future time t+τ. In order to remove the information from the history of *Y*, the dependence between x(t) and y(t+τ) is conditioned on y(t),y(t−ρ),…,y(t−(n−1)ρ). If *Y* is an *n*-dimensional dynamical system, the dimensionality of the condition must contain full information about the system state in *n* components, while single components x(t) and y(t+τ) are able to provide information about the directional coupling, i.e., the causality between the systems X(t) and Y(t).

The CMI of Equation (11) is used for testing the existence of a causal link from *X* to *Y*, denoted as X→Y. The causal link Y→X can be obtained by full analogy with Equation (11), as

(12) $$I\left(y(t);x(t+\tau )|x(t),x(t-\eta ),\ldots ,x(t-(m-1)\eta )\right).$$

However, Paluš [39] warned that, in general, the prediction horizon τ in Equation (11) or (12) cannot well represent a time-delayed coupling. This also was shown by Wibral et al. [40] and they proposed a solution by a simple reformulation of Equation (11) as

(13) $$I\left(x(t);y(t+\tau )|y(t+\tau -1),y(t+\tau -1-\rho ),\ldots ,y(t+\tau -1-(n-1)\rho )\right).$$

### 3.3. Linear-Gaussian CMI

Let us return to an *n*-dimensional normally distributed random variable $X_{1},\ldots ,X_{n}$. Its mutual information $I_{G}\left(X_{1};\ldots ;X_{n}\right)$ is given by Equation (7). If the variables are normalized to zero mean and unit variances, Equation (7) can be simplified as

(14) $$I_{G}\left(X_{1};\ldots ;X_{n}\right)=-\frac{1}{2}\;\sum_{i=1}^{n}\log\left(\sigma_{i}\right),$$

where $\sigma_{i}$ are the eigenvalues of the n×n correlation matrix. Now using Equations (14) and (9) we can express the conditional mutual information (13) using just the eigenvalues of correlation matrices of the relevant variables and call this form of CMI linear or Gaussian CMI estimator.

### 3.4. Liang Information Flow

While the conditional mutual information [33] and the transfer entropy [23] have been proposed for the detection of causality using heuristic arguments, Liang [41] defined an information flow as a general physical notion that can be rigorously derived from first principles. Liang derived the information flow for both deterministic and stochastic dynamical systems using the equations describing the evolution of such systems. For experimental situations when time series are available, however, the underlying equations are not known, Liang was able to derive a concise formula for linear systems with a dependence structure fully described by correlation/covariance matrices.

Let us consider *n* random variables $X_{1},\ldots ,X_{n}$ represented by time series $x_{1}\left(t\right),\ldots ,x_{n}\left(t\right)$, t=1,…,N and define the sample covariance matrix C

$$C_{ij}=\frac{1}{N}\sum_{t=1}^{N}\left(x_{i}\left(t\right)-{\bar{x}}_{i}\right)\left(x_{j}\left(t\right)-{\bar{x}}_{j}\right),$$

where

$${\bar{x}}_{i}=\frac{1}{N}\sum_{t=1}^{N}x_{i}\left(t\right).$$

Using the differenced time series

$${\dot{x}}_{i}\left(t\right)=\frac{1}{\tau }\left(x_{i}\left(t+\tau \right)-x_{i}\left(t\right)\right)$$

we define matrix D as

$$D_{ij}=\frac{1}{N}\sum_{t=1}^{N}\left(x_{i}\left(t\right)-{\bar{x}}_{i}\right)\left({\dot{x}}_{j}\left(t\right)-{\bar{\dot{x}}}_{j}\right).$$

The Liang [41] information flow from $X_{2}$ to $X_{1}$ is

(15) $$T_{2\to 1}=\frac{1}{\det\;C}\sum_{j=1}^{n}\Delta_{j2}D_{j1}\frac{C_{12}}{C_{11}},$$

where $\Delta_{ij}$ are the cofactors of C.

### 3.5. Interventional Causality

All information-theoretic measures for causality detection try to find how much the knowledge of a given variable (say *X*) is helpful to predict the future values of another variable (say *Y*). In the framework of physics, however, if the causal link X→Y exists, one usually expects to observe the consequences of external perturbations of variable *X* on variable *Y* [36,42]. Recently, Baldovin et al., have shown that for a multidimensional linear Markov system one can extract the causal relations among the system components using time correlations as well as the response theory [43]. For this physics based causality they coin the term *interventional* causality and the information based one they call *observational* causality. In order to measure the strength of the interventional causality, one should find the response matrix, as defined in the response theory in statistical physics [44]. Assume that a system with a set of *n* linearly coupled variables $x_{t}=\left\{x_{t}^{\left(1\right)},x_{t}^{\left(2\right)},...,x_{t}^{\left(n\right)}\right\}$, obeys the stochastic dynamics of $x_{t+1}=Ax_{t}+B\eta_{t}$, where *A* and *B* are constant n×n matrices and vector $\eta_{t}$ has independent and identically distributed random components with zeros means and unitary variances. One can calculate the response matrix $R_{\tau }$ using the covariance matrix *C* as follows [43,44]

(16) $$R_{\tau }=A^{\tau }=C_{\tau }C_{0}^{-1}$$

where $C_{\tau }^{ij}=\langle x_{t+\tau }^{i}x_{t}^{j}\rangle$. This indicates that if $R_{\tau }^{ij}\neq 0$, then a causal link exists between the present of $x^{j}$ and the future of $x^{i}$. Baldovin et al. also showed that this formalism is able to discover indirect causation. In fact, they argued that if $R_{\tau }^{ij}=0$ for any τ>0, then no causal relationship exists. However, if $R_{\tau }^{ij}=0$ for τ≤m−1, and $R_{\tau }^{ij}\neq 0$ for τ≥m, then there exists at least a path of length *m* connecting $x^{i}$ with $x^{j}$.

### 3.6. Statistical Evaluation with Surrogate Data

Estimation of conditional mutual information (CMI) or mutual information (MI) from finite time series may result in a spurious conclusion about inferring the direction of coupling. On the other hand, the CMI (or MI) estimates for uncoupled time series may yield a nonzero value. Due to such biases, the absolute values of such quantities may not be informative, and a comparison between the values obtained from observed processes and that from uncoupled processes, which share important properties of the observed ones, is useful. This can be done by a surrogate testing approach, in which one manipulates the original data in a randomization procedure, which preserves some distinct features of the original process [45].

Among various types of surrogate tests, the circular time-shifted surrogates method has been shown to be well adapted for causality calculations [46]. In order to compute the statistical significance of our calculations, we apply this surrogate test. Accordingly, for each time series *X*, we generate 100 independent realizations of time-shifted surrogates as follows: An integer variable *k* is randomly chosen from the interval [0.01,0.99]N, where *N* is the total number of sample points in the series. Then, by moving the first *k* values of X(1),X(2)…X(k) to the end of the time series, we generate the circular time-shifted surrogate series $X^{surr}$ as

(17) $$X^{surr}=\left\{X\left(k+1\right),X\left(k+2\right),\ldots ,X\left(N\right),X\left(1\right),X\left(2\right),\ldots ,X\left(k\right)\right\}.$$

Note that in generating such surrogate series, we preserve the whole statistical structure of the original time series. In our analysis, the (conditional) mutual information values calculated from the original data are compared with the range of values obtained from a set of 100 different realizations of the surrogate series. This means that by calculating the mean and the variance of those 100 surrogate series, one can measure how much the obtained information-theoretic measure obtained from original series differs from that of uncoupled processes.

## 4. Results and Discussion

### 4.1. Causality and Time Delays

**Figure 2:**
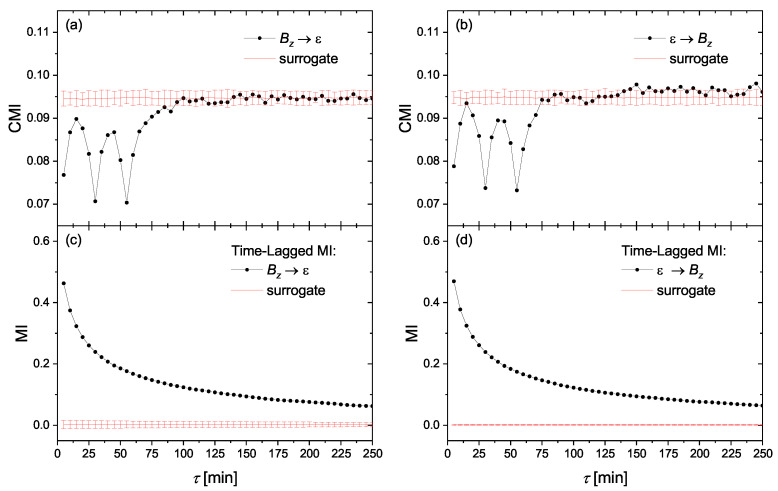
The conditional mutual information (CMI) (Equation (13)) obtained from the time series of Bz and ϵ, for the information flow directions of (**a**) Bz→ϵ and (**b**) ϵ→Bz. The corresponding time-lagged mutual information (MI) between these two variables are represented in (**c**,**d**), respectively. The red lines and error bars represent mean and ±2 standard deviations for a set of 100 circular time-shifted surrogates.

As we mentioned above, in order to find the causality directions as well as the presence of any information transfer delay between the solar wind and the geomagnetic indices, we calculate conditional mutual information among such time series using CMI defined in Equation (13), computed using the equiquantal binning estimator [34]. In Figure 2a,b, we represent the obtained CMI for two time series of Bz and ϵ with ρ=5 and n=3 as the embedding construction parameters. Note that red lines and error bars, respectively, show the mean and ±2 standard deviations (SD) of the corresponding CMI obtained from a set of 100 circular time-shifted surrogates. For the evidence of causality we apply the one-sided test, i.e., CMI is considered significantly positive if its value, obtained from the analyzed data, is distinctively greater than the mean + 2SD of the surrogate values. This criterion ensures that the evidence for causality (a positive CMI value) did not occur by chance, considering also the multiplicity of the tests for a range of time lags. On the other hand, the digression of the CMI values under the surrogate mean–2SD range does not have any evidential meaning and can be understood considering Equation (9) giving CMI as the difference of mutual information functionals. In the surrogate data we destroy all dependence structures, while in the tested data there is no causal information between the two variables, but still there is some nonzero auto-information $I\left(y(t+\tau );y(t+\tau -1),y(t+\tau -1-\rho ),\ldots \right)$ in the effect variable, which is subtracted in Equation (9). Thus we find that Bz and ϵ do not show any causal relationship in both directions. However, this is due to the fact that Bz and ϵ are “synchronized” (mutually dependent), which can be easily inferred [47] from high, significantly positive, values of the time-lagged mutual information (MI) among these two variables, as indicated in Figure 2c,d for $I\left(Bz(t),\epsilon (t+\tau )\right)$ and $I\left(\epsilon (t),Bz(t+\tau )\right)$, respectively. Thus, in our further analysis we only consider Bz as the solar wind driver.

**Figure 3:**
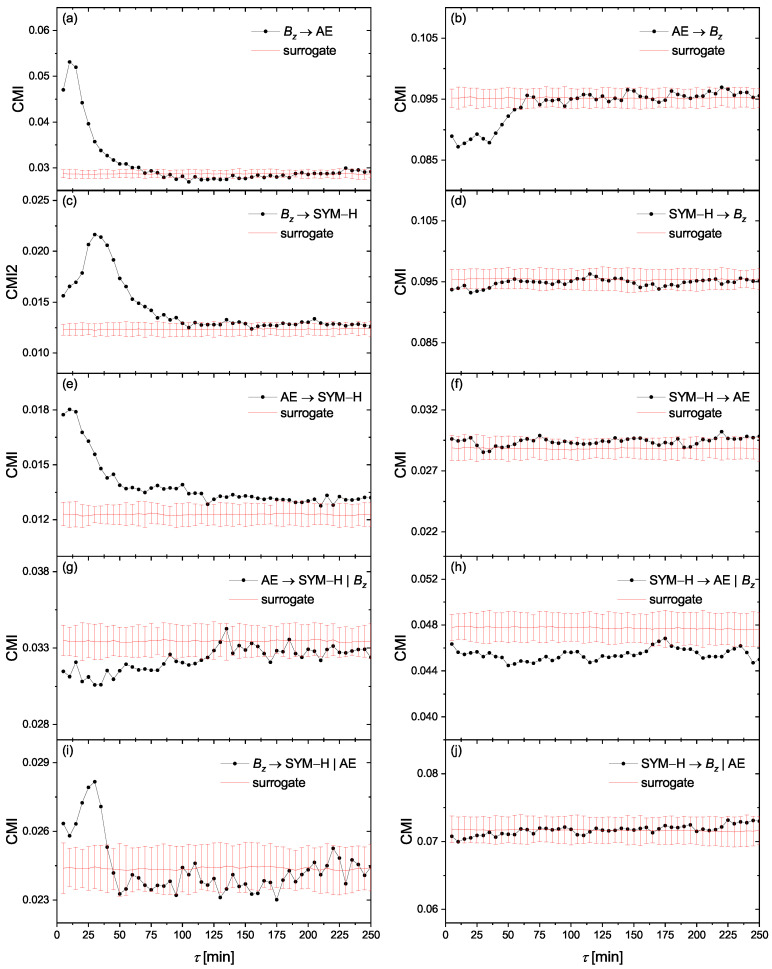
The conditional mutual information (CMI) (Equation (13)) obtained from the time series of Bz, AE and SYM-H, for the information flow directions of (**a**) Bz→ AE, (**b**) AE →Bz, (**c**) Bz→ SYM-H, (**d**) SYM-H →Bz, (**e**) AE → SYM-H, and (**f**) SYM-H → AE. (**g**,**h**) are the information flow directions similar to (**e**,**f**), by taking Bz as the third variable into the condition (see Equation (18)). Analogously, (**i**,**j**) are the information flow directions similar to (**c**,**d**), by taking AE as the third variable into the condition. The red lines and error bars present mean and ±2 standard deviations of CMI for a set of circular time-shifted surrogates.

Accordingly, we represent in Figure 3a,b, the CMI obtained from time series of Bz and AE in order to investigate the impact of the solar wind driver Bz on the geomagnetic observable of AE. As it can be seen from Figure 3a, a strong causal link exists from Bz to AE and also the information transfer takes two sample time steps (10 min). Also, Figure 3b indicates that there is no causal relationship from AE to Bz. Similarly, we plotted CMI for the time series of Bz and SYM-H in Figure 3c,d. Figure 3c shows that a causal relationship also exists from Bz to SYM-H; however, this time the information transfer takes six sample time steps (30 min). Also, no causality is observed for the reverse direction, as indicated in Figure 3d. Our findings confirm that both geomagnetic storms and substorms are driven by the interplanetary magnetic field component Bz, just with different information transfer delays. The response time for the magnetic storms is longer than the time delay between the solar wind energy input and the release of energy in the magnetotail during a substorm (see [48] and references therein) since it takes a considerably long time to inject particles into the ring current region [49].

One of the challenging problems in space weather studies is to find a possible causal relationship between substorms and storms. To check the presence of any information flow between the geomagnetic indices, we plotted in Figure 3e,f the CMI for time series of AE and SYM-H. Figure 3e indicates that a strong causality from AE to SYM-H exists; in other words, substorms drive the geomagnetic storms. Also, this information transfer occurs almost immediately without any delay. Figure 3f also indicates that there is no causality from storms to substorms. Indeed, this finding is in line with some previous studies [15,22,50,51]. However, we show that the observed information transfer in Figure 3e is not a direct causal link since it emerges due to the presence of the common driver Bz. To show this, we should take into account the effects of this common driver in calculating CMI. Accordingly, including Bz as the third variable into the condition, we obtain

(18) $$I\left(\mathrm{AE}(t);\mathrm{SYMH}(t+\tau )|\mathrm{SYMH}(t+\tau -1),Bz(t+\tau -1-\rho ),Bz(t+\tau -1-2\rho )\right).$$

In Figure 3g,h we represent the CMI of Equation (18) obtained from the time series of AE and SYM-H, given Bz. Interestingly, we discover that there is no causality between AE and SYM-H. In fact, by removing the role of the common driver Bz, no information flow exists in both directions between storm and substorm indices of AE and SYM-H. In order to verify this finding, we also search for the possible impact of AE on the observed causal link between Bz and SYM-H, by interchanging variables of Bz and AE in Equation (18). As can be seen in Figure 3i,j, we find that the causal link of Bz→ SYM-H is independent of the AE index. Briefly, our results suggest that the observed causal link from geomagnetic substorms into storms is induced by the common solar wind driver Bz and in fact, there is no causal relationship between substorms and storms, which is in agreement with some previous studies [10,11,14,21].

### 4.2. Linear Mass-Energy Transfer

**Figure 4:**
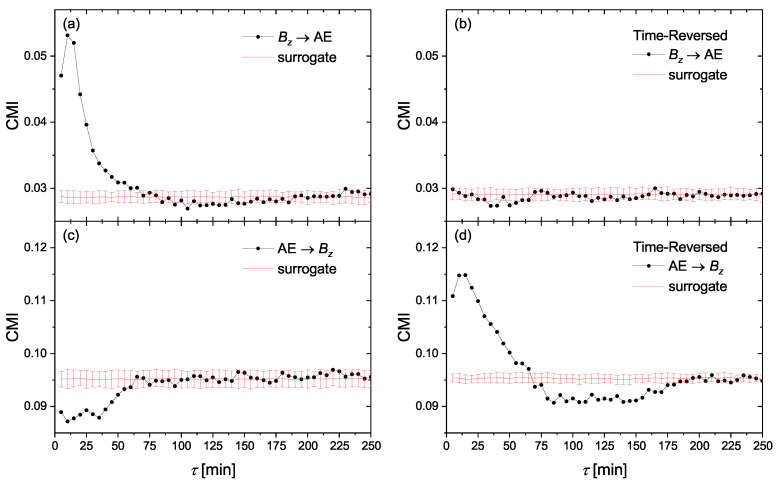
The conditional mutual information (CMI) of Equation (13) for the information flow directions of (**a**) Bz→ AE, (**c**) AE →Bz. (**b**,**d**) are, respectively, the same as (**a**,**c**), but for time-reversed series.

**Figure 5:**
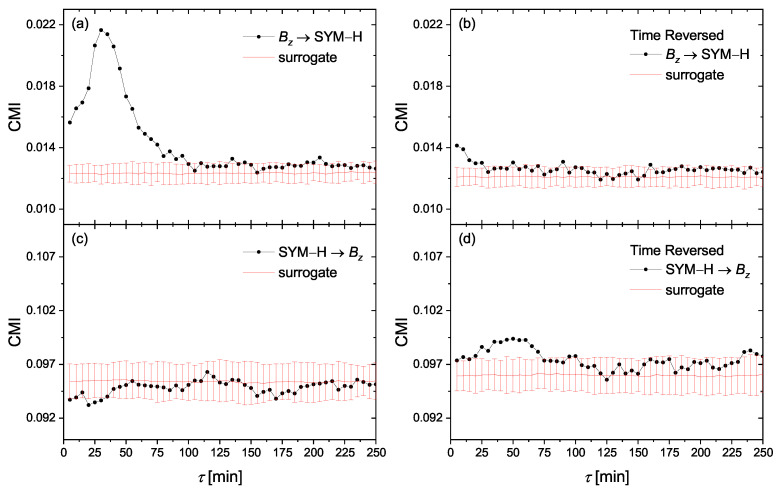
The conditional mutual information (CMI) of Equation (13) for the information flow directions of (**a**) Bz→ SYM-H, (**c**) SYM-H →Bz. (**b**,**d**) are, respectively, the same as (**a**,**c**), but for time-reversed series.

Paluš et al. have shown [52] that the time reversal in causality analysis can help to distinguish between a linear transfer of a time-delayed process and nonlinear interactions of dynamical systems. Indeed, they showed that in linear autoregressive processes with unidirectional causality, when the independent variable X(t) is causing the variable Y(t) by a simple linear, time-delayed term cX(t−τ), the causality direction X→Y is reversed after the time reversal into Y→X. On the other hand, nonlinear dynamical systems violate the Granger causality principle that the cause precedes the effect and the direction of causality is not reversed after the time reversal. In this respect, we investigate the causality relationships for time reversed series. Figure 4 represents causal directions between Bz and AE for original time series ((a) and (b)) and the corresponding time-reversed series ((c) and (d)). Figure 5 is similar to Figure 4, but for SYM-H instead of AE. The plots have the same scale for better comparison. We find that, particularly in the case of Bz and AE, the causal direction is reversed after time reversal, which indicates that a simple linear mass-energy transfer may exist from the solar wind driver Bz into the geomagnetic indices of AE and SYM-H. The case of Bz–SYM-H is more complicated and will be explained below.

**Figure 6:**
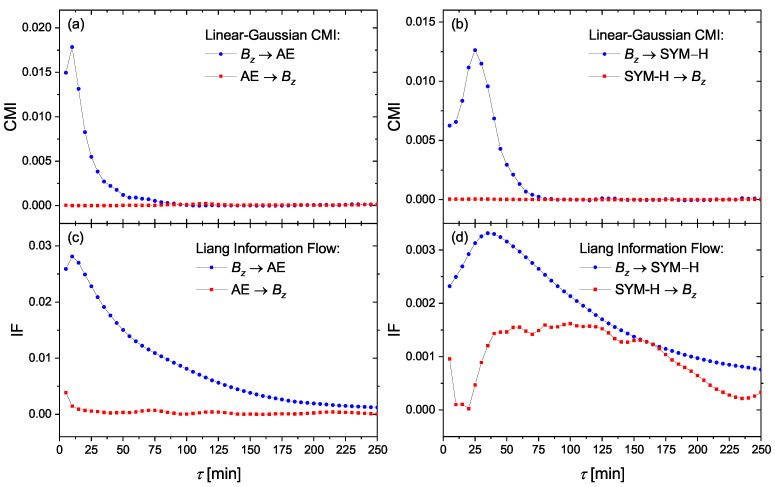
The causality relationships between the solar wind driver Bz and the geomagnetic observables of AE and SYM-H, using linear-Gaussian CMI (**a**,**b**) and Liang information flow (**c**,**d**).

In order to confirm the hypothesis of a linear information transfer we recompute the CMI functionals in their version derived for Gaussian processes when the (conditional) mutual information can be expressed using linear cross-correlations of the studied variables (see Section 3.3). In this respect, we demonstrate in Figure 6a,b that the causal relationships obtained from the linear-Gaussian CMI between the driver Bz and the geomagnetic indices of AE and SYM-H, respectively, are equivalent to those obtained by the CMI estimator based on probability distribution functions (Figure 3), which reflects general (i.e., also nonlinear) dependence structures. For simplicity we do not present the significance tests in these cases, since the information flow in the direction from Bz to the geomagnetic indices AE and SYM-H, respectively, is distinctively positive while the information flow in the opposite direction is nearly zero. Figure 6c,d represent the causality relations similar to (a) and (b), but using the Liang information flow according to the Formula (15) derived for linear processes. This measure admits some information flow in the direction towards Bz—probably higher dimensional embedding would be necessary to support the hypothesis of the unidirectional causality. However, the Liang information flow, the concept entirely independent of CMI (transfer entropy) or the Granger causality concept, confirms the dominant information flow in the direction from Bz to AE and SYM-H. For the information transfer from Bz to AE the Liang information flow also confirms the information transfer delay of 10 min, while the lag-dependence for the information transfer from Bz to SYM-H has a broader peak giving the information transfer delay in the range 25–35 min. This might indicate not only larger time delay, but probably also more complicated structure (multiple lags) in the causal influence of Bz to SYM-H. The latter can also explain weaker but bidirectional information transfer after the time reversal (Figure 5). More complicated AR processes (higher order, multiple time lags, or non-Gaussian innovations) do not simply reverse the causality, but bidirectional causality is observed after the time reversal. (See Chvosteková et al. [53].)

**Figure 7:**
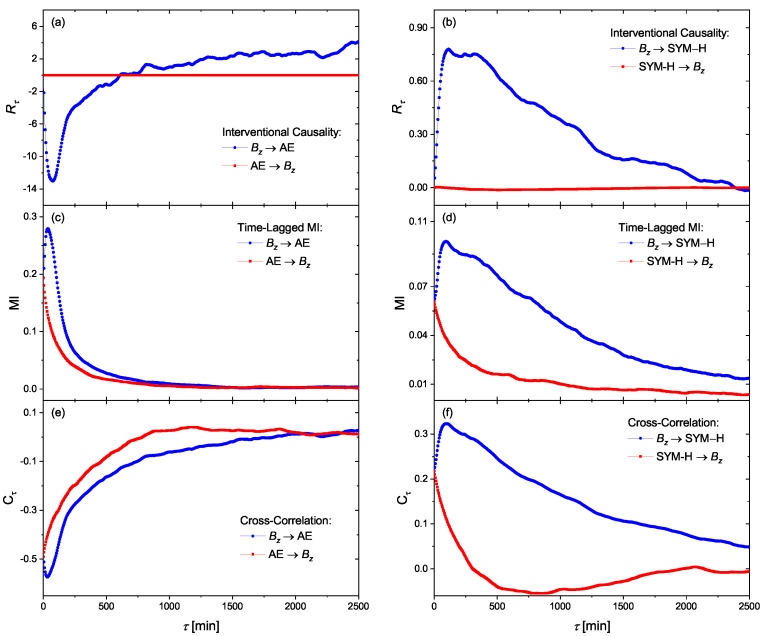
The response matrix $R_{\tau }$ elements for the solar wind driver Bz and the geomagnetic observables of (**a**,**b**) SYM-H. The corresponding time-lagged mutual information ((**b**,**c**)) as well as the cross-correlation (**e**,**f**) for Bz versus AE (**a**,**c**) and Bz versus SYM-H (**b**,**d**) are shown for better comparison.

Finally, by assuming a linear mass-energy transfer from solar wind into a geomagnetic environment, we investigate the interventional causal links between Bz and the indices of AE and SYM-H, as indicated in Figure 7a,b, respectively. As we expected, a strong (weak) causal link exists from Bz into AE (SYM-H). However, the time-delays in causality relations are not the same as what we previously obtained based on information transfer. We argue here that the interventional causality, which is based on the response theory and covariance (correlation) between variables, is not capable of detecting time-delayed causation. In fact, such different time delays observed in this method are the consequence of its cross correlation nature (see Equation (16)). To show this, we plotted the time-lagged mutual information in Figure 7c,d, as well as the normalized cross-correlation in Figure 7e,f, between these variables. As expected, we observe nearly the same time delays as in (a) and (b). On the other hand, the negative response of AE to Bz is observed in the correlation indicated in Figure 7e. Our findings based on interventional causality demonstrate that AE is strongly driven by Bz via a (negative) linear impact. On the other hand, the solar wind driver Bz, drives the geomagnetic storms of SYM-H by a weaker (positive) nearly linear information transfer.

## 5. Conclusions

An information-theoretic approach to causality detection was used in order to contribute to the understanding of how the magnetosphere–ionosphere system of the Earth responds to solar activity and to interplanetary medium conditions. Conditional mutual information, also known as transfer entropy, which generalizes the Granger causality concept for nonlinear systems, was applied to time series of the vertical component of the interplanetary magnetic field Bz and the Perreault–Akasofu coupling function ϵ characterizing the solar wind and interplanetary medium conditions and geomagnetic activity indices AE as a substorm index and SYM-H as a magnetic storm index. A unidirectional causality, or information flow, from the solar wind to the geomagnetic indices was demonstrated. In particular, Bz causes AE with the information transfer delay of 10 min, and Bz causes SYM-H with the information transfer delay of about 30 min. In bivariate CMI analysis also, AE causes SYM-H; however, after taking CMI conditionally on Bz, no causal relation between AE and SYM-H can be detected. Thus, the causal influence of substorms on magnetic storms, in particular the causality AE→SYM-H reported, for example, by Stumpo et al. [22] is in fact a secondary relation induced by the common cause, Bz.

The problem of three or more variables involved in causality analysis has recently been intensively discussed not only in the context of causal graphs or directed networks estimated from multivariate time series (see, e.g., [54] and the already cited application in space weather [21]) but also in the study of higher-order interactions, which, tackled using the tools of information theory, requires the decomposition into unique, redundant and synergistic information (see [55,56] and the related Entropy Special Issue introduced by [57]).

Studying causality after time reversal indicates that the detected causal relation, although observed in nonlinear, out of equilibrium processes, can be explained by a linear, time-delayed information transfer. In order to support this conjecture we computed three different causality measures derived for linear systems: CMI derived for Gaussian processes, Liang information flow [41] in its version for linear systems, and interventional causality derived for linear Markov systems using time correlations as well as the response theory [43]. All these methods confirmed the information flow from the solar wind to the geomagnetic indices and the linear CMI and Liang information flow, as well as the information transfer delays of 10 min for the relation Bz→AE, and 25–35 min for the relation Bz→SYM-H. The interventional causality peaks in larger time delays are consistent with cross-correlations or time-lagged mutual information. It is known, however, that the latter as well as CMI/transfer entropy in its standard definition are not reliable tools for determining the information transfer delays [40].

We believe that the presented results contribute to a better understanding of solar wind-magnetosphere–ionosphere interactions as well as to modeling and predictions of space weather events. This study adds further compelling evidence to previous studies (e.g., [21,58,59,60,61,62,63]), highlighting the great potential of information-theoretic approaches to contribute in the development of Space Weather diagnostics and tackle contemporary research problems in Space Physics.

## Data Availability

All data used in this study are publicly available, see Section 2 Data Description.
